# Classification of Pancreatic Ductal Adenocarcinoma Using MALDI Mass Spectrometry Imaging Combined with Neural Networks

**DOI:** 10.3390/cancers15030686

**Published:** 2023-01-22

**Authors:** Frederic Kanter, Jan Lellmann, Herbert Thiele, Steve Kalloger, David F. Schaeffer, Axel Wellmann, Oliver Klein

**Affiliations:** 1 Institute of Mathematics and Image Computing, Universität zu Lübeck, 23562 Luebeck, Germany; 2 Fraunhofer Institute for Digital Medicine MEVIS, 23562 Luebeck, Germany; 3 Department of Pathology and Laboratory Medicine, University of British Columbia, Vancouver, BC V6T 1Z4, Canada; 4 Pancreas Centre BC, Vancouver, BC V5Z 1G1, Canada; 5 Division of Anatomic Pathology, Vancouver General Hospital, Vancouver, BC V5Z 1M9, Canada; 6 Institute of Pathology, Wittinger Strasse 14, 29223 Celle, Germany; 7 BIH Center for Regenerative Therapies, Berlin Institute of Health at Charité-Universitätsmedizin Berlin, 13353 Berlin, Germany

**Keywords:** MALDI mass spectrometry imaging, pancreatic ductal adenocarcinoma, neural-network models

## Abstract

**Simple Summary:**

Pancreatic ductal adenocarcinoma (PDAC) accounts for more than 90% of all pancreatic malignancies, and has a generally poor prognosis. Although it is the most common neoplastic disease of the pancreas, differential diagnosis is hindered by the lack of accurate and reliable diagnostic assays. Identification of molecular signatures for PDAC diagnosis offers a solution to improve the clinical and patient management. However, comprehensive omics profiling is time- and relatively cost-intensive and limited by tissue heterogeneity. Thus, it is not implemented in clinical routine. In this study, we investigate the feasibility of MALDI-MSI in combination with a neural-network-based analysis for accurate classification of pancreatic-ductal-adenocarcinoma patients. We provide evidence of the usefulness of this technology to support PDAC assessment which is promising for pathological aid.

**Abstract:**

Despite numerous diagnostic and therapeutic advances, pancreatic ductal adenocarcinoma (PDAC) has a high mortality rate, and is the fourth leading cause of cancer death in developing countries. Besides its increasing prevalence, pancreatic malignancies are characterized by poor prognosis. Omics technologies have potential relevance for PDAC assessment but are time-intensive and relatively cost-intensive and limited by tissue heterogeneity. Matrix-assisted laser desorption/ionization mass spectrometry imaging (MALDI-MSI) can obtain spatially distinct peptide-signatures and enables tumor classification within a feasible time with relatively low cost. While MALDI-MSI data sets are inherently large, machine learning methods have the potential to greatly decrease processing time. We present a pilot study investigating the potential of MALDI-MSI in combination with neural networks, for classification of pancreatic ductal adenocarcinoma. Neural-network models were trained to distinguish between pancreatic ductal adenocarcinoma and other pancreatic cancer types. The proposed methods are able to correctly classify the PDAC types with an accuracy of up to 86% and a sensitivity of 82%. This study demonstrates that machine learning tools are able to identify different pancreatic carcinoma from complex MALDI data, enabling fast prediction of large data sets. Our results encourage a more frequent use of MALDI-MSI and machine learning in histopathological studies in the future.

## 1. Introduction

Pancreatic ductal adenocarcinoma (PDAC) accounts for more than 90% of all pancreatic malignancies, and has generally a poor prognosis [1]. With a 5 year overall-survival of less than 8%, PDAC is the fourth most frequent cause of cancer-related deaths worldwide [2]. Projections indicate that the number of PDAC diagnoses as well as PDAC-related deaths will more than double in the next decade in the United States [3] and in European countries [4]. This is due to lifestyle habits such as alcohol and tobacco abuse, which are known to increase the risk of several other cancers and also seem to play a role in the development of PDAC [4,5,6,7,8]. Pancreatic ductal adenocarcinoma (PDAC) and ampullary carcinoma (AC) are gastrointestinal cancers with overlapping clinical symptoms [9]. Although several studies hypothesize that pathogeneses and molecular composition are different, the clinical regime and therapy remain similar [10]. Moreover, existing studies demonstrate that the 5 year overall-survival-rate of patients with PDAC is lower in comparison with patients with AC [11]. Pancreatic ductal adenocarcinoma of the pancreas and ampullary carcinoma arise in close proximity to each other [12]. As a result, differential diagnosis of PDAC remains clinically challenging. Differential diagnosis is hindered by the lack of accurate and reliable diagnostic assays. Identification of molecular signatures for PDAC diagnostics offers the possibility of improving the clinical and patient management. However, common tissue-based proteomic and genomic techniques require large amounts of homogenized tissue material, which does not enable a direct correlation of molecular alterations with tissue histology.

Matrix-assisted-laser-desorption/ionization (MALDI) imaging technology combines common mass spectrometry with histological approaches. This technique is suitable for analyzing molecules (e.g., metabolites, proteins, peptides, lipids and glycans) and their spatial distribution in a single tissue-section in an unsupervised and label-free manner [13,14,15,16]. MALDI mass spectrometry imaging (MALDI-MSI) enables the high-throughput determination of spatial molecular-signatures in a clinically acceptable period and at relatively low cost, in comparison to other omics technologies. This provides new capabilities to classify different patient subgroups and even supports prediction of disease progression and/or resistance development.

In previous studies, MALDI-MSI was applied to in situ proteomic analysis of preneoplastic lesions in pancreatic cancer in genetically engineered mice [17]. In the study, intraepithelial neoplasia (PanIN) and intraductal papillary mucinous neoplasm (IPMN) could be discriminated from normal pancreatic tissue and early pancreatic ductal adenocarcinoma. Further studies have shown differences in the chemical structure of phospholipids and their distribution patterns in human pancreatic islets with intra-islet spatial resolution using MALDI-MSI [18]. Besides the investigation of the underlying mechanism, several proof-of-concept studies demonstrate the potential of MALDI-MSI in combination with machine learning algorithms to identify peptide signatures of prognostic relevance in pancreatic cancer [19,20].

In recent years, neural networks gained great popularity in many machine learning tasks such as image and speech recognition [21], image segmentation [22], and various classification tasks. Convolutional neural networks especially outperform many classical machine-learning approaches [23]. Neural networks are able to approximate highly complex decision functions, through their layer structure. Each layer consists of so-called neurons, which themselves contain weights and a nonlinear gating function. During training, the weights are tuned to solve the task at hand, which typically goes hand in hand with intrinsic learning, a meaningful feature-representation of the given data [24]. In the context of MALDI-MSI, machine learning algorithms have been used less frequently. Most studies make use of statistical methods, such as hierarchical clustering [25] or linear discriminant analysis (LDA). The high-dimensional nature of MALDI-MSI spectra often prohibits applying machine learning directly to the raw spectrum. Thus, strategies for feature selection or dimension reduction are employed to enable machine learning tools to be used. The most commonly used techniques are peak picking [26], principal component analysis (PCA), and non-negative matrix factorization (NMF) [27].

**Figure 1:**
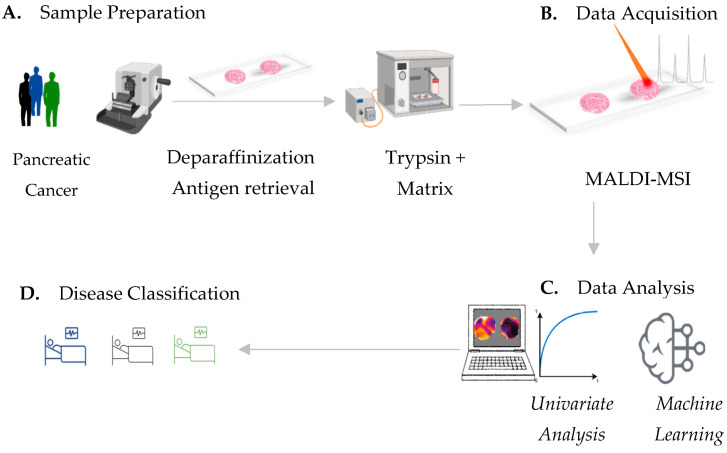
MALDI-MSI workflow for pancreatic-cancer-tissue assessment. (**A**) Pancreatic-cancer-tissue sample preparation includes deparaffinization, antigen retrieval followed by tryptic digestion and matrix application. (**B**) In situ determination of spatial mass to charge (*m/z*) signatures by means of MALDI mass spectrometry imaging. (**C**) Univariate testing of MALDI-MSI-derived data provides single discriminative *m/z* values, which can potentially identify distinct tissue types. Machine learning by neural-network strategies provides (**D**) an accurate classification of pancreatic-cancer types, which is potentially sufficient for clinical routine.

In this proof-of-concept study, we investigate the feasibility of a neural network-based analysis of MALDI-MSI data for accurate classification of pancreatic ductal adenocarcinoma (Figure 1). We explicitly rely on the inherent feature-selection capability of neural networks, and evaluate the feasibility of feeding the full-scale spectral data to the classification methods in order to reduce the problem-specific modeling overhead as well as human interaction, and allow all available data to be used.

## 2. Materials and Methods

### 2.1. Patient and Sample Cohort

Tissue microarrays (TMAs) from formalin-fixed paraffin-embedded tissue from patients diagnosed with exocrine pancreatic cancer, in particular pancreatic ductal adenocarcinoma (PDAC) and ampullary carcinoma (AC) were prepared at the University of British Columbia Research (Table 1). The use of these samples is covered by ethical approval from the University of British Columbia Research Ethics Board H22-00073. Besides PDAC and AC, the investigated cohort included tissue material of other pancreatic cancer types, which are: acinar cell carcinoma, carcinoma NOS, benign chronic pancreatitis, intraductal papillary-mucinous carcinoma-invasive, intraductal papillary-mucinous carcinoma-noninvasive, mucinous cystic neoplasm-noninvasive, mucinous noncystic carcinoma, neuroendocrine tumor, pseudo-papillary tumor, serous cystadenoma, and signet-ring cell carcinoma.

### 2.2. MALDI-MSI

Formalin-fixed paraffin-embedded (FFPE) tissue sections (tissue microarrays) were prepared as described before [28]. Briefly, 6-µm thick tissue-sections were mounted onto conductive glass slides, coated with indium tin oxide (Bruker Daltonik GmbH, Bremen, Germany). Sections were preheated to 80 °C for 15 min, followed by paraffin removal, and heat-induced antigen retrieval. Trypsin solution (20 µg Modified Porcine Trypsin in 800 µL digestion buffer (20 mM ammonium bicarbonate with 0.01% glycerol) was applied by an automated spraying device (HTX TM-Sprayer, HTX Technologies LLC, ERC GmbH, Riemerling, Germany), at 30 °C. The tryptic digest was performed in a humidity chamber for 2 h, at 50 °C. Matrix solution (7 g/L a-cyano-4-hydroxycinnamic acid in 70% acetonitrile and 1% trifluoroacetic acid, at 75 °C) was applied using an HTX TM sprayer. A RapifleX MALDI Tissuetyper with flexImaging 5.1 and flexControl 3.0 software (Bruker Daltonik GmbH, Bremen, Germany) was used in positive-ion reflector mode, detection range of 800–3200 *m/z*, 500 laser-shots per spot, a sampling rate of 1.25 GS/s (gigasamples per second) and a raster width of 50 µm for MALDI-MSI data acquisition. External calibration was carried out using a peptide calibration standard (Bruker Daltonik GmbH). After matrix removal, TMA sections were stained with hematoxylin and eosin, for histology. Tumor regions were digitally annotated in the QuPath open-source software by a pathologist, and transferred into SCiLS Lab software (Version 2019c Pro, Bruker Daltonik GmbH).

### 2.3. Processing of MALDI-MSI Data

MALDI-MSI raw data were converted to SCiLS Lab base data .sbd file and SCiLS Lab extended file .slx format using SCiLS Lab software version 2019c Pro (Bruker Daltonik GmbH). Data were set to total ion count without baseline removal. Patient tissue cores were categorized into ductal adenocarcinoma and non-PDAC (AC+ other pancreatic cancer types) attributes, to split the data into independent data sets (different tumor or patient-characteristics) for analysis. For peak detection and alignment, a standard segmentation pipeline (SCiLS Lab software) was used with the following parameters: width = 0.2 Da, maximal interval processing, total ion-count TIC normalization, medium noise reduction and no smoothing (Sigma: 0.75) [29,30].

### 2.4. Univariate Statistical Analyses

Supervised receiver-operating-characteristic (ROC) analyses were applied to identify *m/z* values, which are discriminative between tumor tissue regions of pancreatic ductal adenocarcinoma (PDAC) and ampullary-carcinoma (AC) tumor tissue regions. Area-under-the-curve values (AUC) close to 0 and 1 indicate that *m/z* values (peptides) are discriminatory. A comparable number of spectra must be used for the ROC analyses, and 10,000 spectra were randomly selected per group. Finally, *m/z* values with an AUC > 0.7 or <0.3 and a *p*-value < 0.001 (Wilcoxon rank-sum test) were selected as discriminative markers.

### 2.5. Protein Identification by Electrospray Ionization Tandem Mass Spectrometry

In order to identify the proteins corresponding to MALDI-MSI-derived *m/z* values (peptide), bottom-up liquid chromatography-based mass spectrometry (LC MS/MS) was performed on adjacent tissue sections, as previously described [26,31]. Briefly, tissue deparaffinization, antigen retrieval and tryptic digest were performed, as for the MALDI-MSI analyses. Using 40 µL of 0.1% trifluoroacetic acid, peptides were extracted from the tissue section. The peptide solution was desalted and purified using a ZipTip^®^ C18, following the manufacturer instructions. Eluates were vacuum concentrated (Eppendorf^®^ Concentrator 5301, Eppendorf AG, Hamburg Germany) and reconstituted separately in 20 µL 0.1% trifluoroacetic acid. A total of 2 µL eluate were injected into a NanoHPLC (Dionex UltiMate 3000, Thermo Fisher Scientific, Waltham, MA, USA) coupled with an ESI-QTOF mass spectrometer (Impact II™, Bruker Daltonic GmbH, Bremen, Germany). All raw spectra from the MS/MS measurement were converted to Mascot generic files (.mgf) using the ProteinScape software. Mascot search engine (version 2.4, MatrixScience; London, UK) and UniProt database were used to analyze mass spectra. The search was performed with the following set of parameters: (i) taxonomy: human; (ii) proteolytic enzyme: trypsin; (iii) peptide tolerance: 10 ppm; (iv) maximum of accepted missed cleavages: 1; (v) peptide charge: 2+, 3+, 4+; (vi) variable modification: oxidation (M); (vii) MS/MS tolerance: 0.8 Da; and (viii) MOWSE score > 25.

The comparison of MALDI-MSI (Supplementary Table S1) and LC−MS/MS *m/z* values (Supplementary Table S2) required the identification of more than one peptide (mass differences < 0.3 Da). The peptides with highest MOWSE peptide score and smallest mass differences between MALDI-MSI and nanoLC-MS/MS data were accepted as correctly identified.

### 2.6. Model Architectures for PDAC Classification

In order to classify PDAC, several neural network-based classifiers were employed. Firstly, a 2-layer residual network with skip connections between each layer, where the input of the proceeding layer was passed unmodified to the subsequent layer as additional input. In the following, this model is denoted as Residual.

Secondly, an encoder-only variant of the Transformer architecture [32] was implemented. The size of the attention matrices used in this model is *n* × *n,* where *n* denotes the sequence length. As each spectrum consists of several thousand data points, applying a Transformer to the full-scale sequence is unfeasible; therefore, the sequence length was reduced using a pooling layer with a kernel-size of 4, based on each peak consisting of 3 individual data points, before passing the input to the encoder. We refer to this architecture as Transformer-1/2.

All models were implemented using the PyTorch (version 1.3.1) framework, and the trainable weights were initialized by randomly selecting values from a truncated normal distribution. Experiments were conducted using the Adam optimizer and rectified-linear-units (ReLU) activation functions. Different hyperparameters such as learning rate, batch size and kernel size were tested. The configurations resulting in the overall best performance on the test set for each architecture are shown in Table 2.

### 2.7. Dataset Design

The data points were converted to NumPy arrays using the NumPy toolbox and a Python-based development environment. We kept the full mass range and stored the spectral data and corresponding label and coordinate information in HDF5 format. A custom tool was used to manually assign spectra to tissue samples. Patient tissue samples with fewer than 20 spectra were excluded, and spectra were normalized to unit median.

The TMA was randomly split into three subsets, which can be seen in Supplementary Figure S1. For both classes (Ductal, non-PDAC), around 70% of the data were used for training the machine learning algorithms, further divided into a training dataset (50% of patient samples) and a validation dataset (20%). The remaining 30% were used as a test dataset to evaluate the classification performance and were not used during the training phase.

We applied 3-fold cross-validation to create distinct data sets for training by repeating the random-splitting process three times (Dataset 1–3). The patient- and spectra-distributions in the training, validation, and test datasets varied slightly among different splits, due to the assignment process. The splitting was performed by selecting full patient core-tissue samples randomly without replacement, and assigning them and all associated spectra to one of the three datasets until the desired size was reached (Table 3, Supplementary Figure S1).

### 2.8. Filtering of Noise Spectra

We implemented a filter to remove spectra with little or no relevance, based on an informativeness score. The informativeness of each spectrum was measured in terms of the number of peaks greater than the variance within the spectrum. We considered a spectrum informative whenever the number of such peaks exceeded a predefined threshold. We restricted the range of data points to evaluate the first 60% of data points in a given spectrum, since there were few peaks present in higher Dalton ranges. We provide a visual interpretation of our measure of informativeness in Supplementary Figure S2.

### 2.9. Classification

Following the preprocessing steps above, the spectra and (during training) class-label information were directly passed to the neural networks. All network classifiers were trained on batched single spectra from the training data-set, monitoring the performance on the validation data-set. The model with the highest accuracy score on the validation data-set was selected to evaluate the performance on the unseen test-data.

Accuracy, sensitivity, and specificity metrics were computed on the unseen test-data in order to measure the performance for each classifier. This evaluation was performed on an individual spectrum level, counting the number of correctly classified spectra to determine the metric scores. In addition, a majority voting-strategy was employed to assign one class to each patient, based on the classes of all associated spectra in that patient’s sample, and metrics were also evaluated on the patient level.

All metrics were averaged over the three different data splits, described in Section 2.7. All experiments were performed on a 2 × 6-core Intel Xeon Gold 6128 CPU @ 3.40 GHz with 24 logical cores and 3× GeForce RTX 2080 Ti GPUs with 11 GB of memory each.

## 3. Results

### 3.1. Acquisition of MALDI-MSI Data

**Figure 2:**
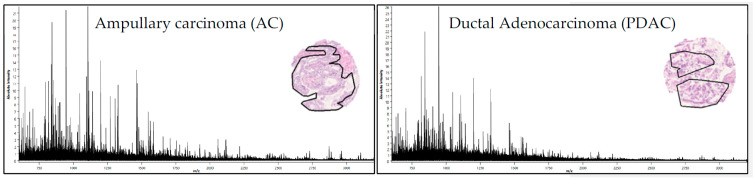
MALDI-MSI derived signature. Average spectra (435 *m/z* values) from AC (**left**) and PDAC (**right**) tumor regions (black line in the H&E staining of representative tissue cores).

We evaluated the feasibility of MALDI-MSI to classify pancreatic ductal adenocarcinoma. Tissue samples were diagnosed from 446 patient-derived tumor-tissue specimens categorized as ductal adenocarcinoma patient (*n* = 260), non-PDAC (*n* = 186, including 103 ampullary carcinoma). The analyzed tumor-tissue-regions underwent peptide signature extraction, which resulted in 435 aligned *m/z* values in the mass range *m/z* 800–3200 Da for tryptic peptides. Representative average spectra from AC (the most common type in the non-PDAC group) and PDAC tissue sections (tumor region) are shown in Figure 2.

### 3.2. Discriminative m/z Values between Pancreatic Ductal Adenocarcinoma and Ampullary Carcinoma

**Figure 3:**
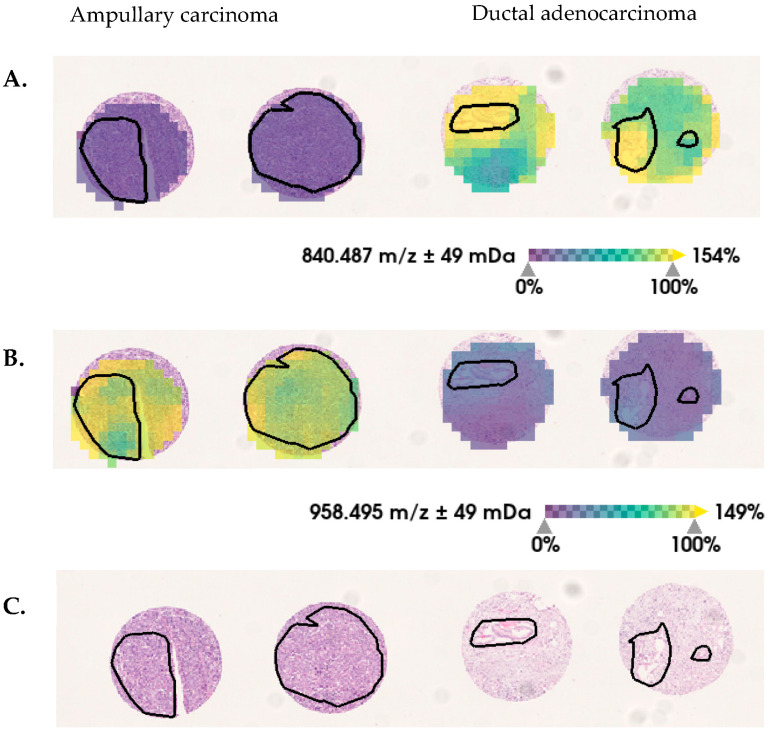
Selected discriminative *m/z* values between ductal-adenocarcinoma and ampullary-carcinoma pancreatic tumor tissue. In tumor regions (black lines), the relative intensity distribution of (**A**) *m/z* 840 Da are decreased and (**B**) *m/z* 958 Da are increased in ampullary carcinoma, in comparison with ductal adenocarcinoma. (**C**) Hematoxylin and eosin (H&E) staining is shown, for orientation.

As pancreatic ductal adenocarcinoma and ampullary carcinoma arise in close proximity to each other, a differential diagnosis of PDAC remains clinically challenging. In order to demonstrate that the MALDI-MSI derived *m/z* values in PDAC are biologically relevant, we performed a univariate pairwise test (ROC) between PDAC and AC. Univariate analysis of MALDI-MSI data was used to determine single *m/z* locations that are discriminative between pancreatic ductal adenocarcinoma and ampullary carcinoma tissue (tumor regions). Receiver-operator-characteristic (ROC) analysis was applied to the total 435 aligned *m/z* peaks from tumor-region areas of tissue sections from PDAC and AC (pairwise comparisons). The intensity distributions of 131 *m/z* values could be identified as discriminative between PDAC and AC (AUC values of >0.7 or <0.3; *p* < 0.001). Representative selections are shown in Figure 3.

### 3.3. Discriminative Proteins Identified from Pancreatic Carcinoma Tissues

**Figure 4:**
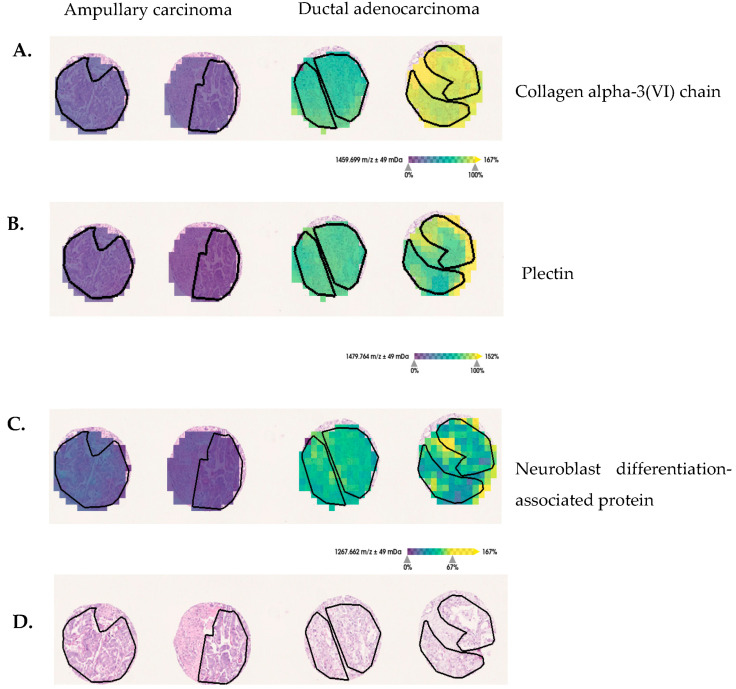
Discriminative protein markers for pancreatic-ductal-adenocarcinoma and ampullary-carcinoma tissue sections. (**A**) The *m/z* 1459 Da assigned to COL6A3, (**B**) *m/z* 1479 Da assigned to PLEC and (**C**) *m/z* 1267 Da assigned to neuroblast differentiation-associated protein show increased intensity-distributions in ductal adenocarcinoma, in comparison with ampullary-carcinoma tumor regions (black lines delineate tumor-border area). (**D**) For orientation, hematoxylin and eosin (H&E) staining are provided.

MALDI-MSI-derived *m/z* values were identified using complementary nanoLC- MS/MS (Supplementary Table S2). Three proteins, corresponding to 7 MALDI-MSI derived *m/z* values (Table 4), fulfilled the criteria to be assumed as identified [26]. The *m/z* values (peptides; Table 4) from PLEC, AHNAK and COL6A3 proteins show significantly higher intensity-distributions in the tumor region of ductal adenocarcinoma, in comparison with ampullary carcinoma (Figure 4). The PLEC protein drives proliferation, migration and invasion in PDAC [33] and is a potential marker for identifying preinvasive lesions [34]. The AHNAK protein is involved in cell proliferation and migration, and thus may affect PDAC outcomes [35]. The COL6A3 protein is described in tissue as well as serum, and is a potential prognostic factor for pancreatic adenocarcinoma [36,37]. These findings support potentially interesting and plausible biological roles in pancreatic ductal adenocarcinoma.

However, the highest AUC is 0.7, which needs to be improved to support PDAC assessment in a clinical setting. Mc Combie et al. [38] demonstrated the multivariate statistical treatment of MALDI imaging-data initially. Multivariate approaches are advantageous in MSI, since they involve the complete mass-spectral information, reduce data dimensions, and obtain differences that are impossible to detect using univariate tests [39,40,41]. More sophisticated machine-learning algorithms aim at revealing more of the intrinsic information hidden in the data by exploring statistical correlations, retrieving data subsets with spectra similarities as well as applying supervised classification methods.

### 3.4. Pancreatic-Ductal-Adenocarcinoma Classifier Identification by Using Neuronal-Network-Evaluation Strategies

In order to increase prediction accuracy of PDAC, we explore the feasibility of MALDI-MSI combined with neuronal-network-evaluation strategies. To reduce the impact of imbalanced training data for convolutional neural networks [42], the classification was performed for PDAC (*n* = 260) against all non-PDAC tissues (*n* = 186, which includes 103 AC and 86 other pancreatic cancer types). The parameter search for all models was carried out using a random-search routine [43]. For the Residual and Transformer models, a high sensitivity to the choice of learning was observed on this data set; other parameters such as batch size and number of epochs did not show a similarly pronounced effect. Lower learning-rates result in the reported accuracies, whereas higher learning-rates hinder convergence during training. Different network depths were tested, but experiments showed that increasing the layer count ultimately harms the overall classification accuracy. Filtering out noise spectra as described in Section 2.8, resulted in the exclusion of approximately 13% of spectra (2183 measurements), resulting in a 3% increase in prediction accuracy for all tested models. The filter was applied to the whole data set before the creation of subsets for training and testing.

Interestingly, some of the samples were consistently labeled incorrectly, across the different model architectures. These samples may express low tumor-cell content and be dominated by the morphological structures of the tissue. Furthermore, the variance within the second class Non-PDAC is high, with multiple subtypes, which may express different spectral patterns. Neural networks tend to work better if the number of features (spectrum size) is relatively small, compared to the number of samples in the data set. However, further investigations with larger cohorts are needed to confirm this trend. Both the Residual model and the Transformer model achieve an average accuracy of 80% on the spectral and patient level over all splits. The highest accuracy on a single split is achieved by the Residual model, with a prediction accuracy of up to 86% on the spectral level and 86% on the patient level. The performance of the Transformer model on the same split is less accurate, with 85% on the spectral level and 86% on the patient level (see Table 5). The attention mechanism in the Transformer architecture does not improve prediction accuracy, compared to the skip connections in the Residual network.

The sensitivity of the Residual network for the ductal histotype is 0.79 on the spectral level and 0.82 for patient prediction. Similarly, the prediction sensitivity of the Transformer model is 0.81 (spectrum) and 0.83 (patient) for the ductal class. The Transformer model achieves a 7% gain on sensitivity compared to the Residual model. Due to the nature of the given two-class problem, the specificity of the Transformer model is 2% lower than the Residual specificity for the ductal class on the patient level (see Table 6).

## 4. Discussion

MALDI-MSI combines spatial molecular (mass-spectrometric) analysis with conventional histological tissue-assessment. This technology enables the simultaneous analyzing of the spatial distribution of hundreds of *m/z* values without prior knowledge (label-free). MALDI-MSI is performed in high-throughput format (less than 5 min/mm^2^ analysis time) with relatively low consumable cost (less than EUR one hundred per glass-slide). Tissue microarrays can be used to transfer up to 100 samples onto a single slide, again reducing the cost per patient-sample. These advantages make MALDI-MSI promising for identifying biomarker signatures and exploring tumor complexity in a clinically relevant format. In the presented study, we were able to demonstrate that the MALDI-MSI analysis results in biologically relevant *m/z* values to discriminate AC and PDAC by using univariate statistical analysis in combination with complementary nanoLC-MS/MS. The statistical analysis (receiver-operating-characteristic; ROC) results in 131 discriminative *m/z* values between pancreatic ductal adenocarcinoma and ampullary adenocarcinoma. Using complementary nanoLC-MS/MS, MALDI-MSI-derived *m/z* values could be assigned to three discriminative proteins: PLEC, Collagen CO6A3, and AHNK. However, direct identification of proteins, from which the *m/z* values (peptides, acquired by MALDI-MSI) stem, remain limited to only a few abundant proteins.

Recent studies have shown that high-resolution MSI data combined with microproteomics (high-resolution mass spectrometry) can be a valuable tool for protein assignment with high mass-accuracy and spatial specificity [44,45,46]. As a result, this strategy is a promising candidate for exploring potentially disease-causing protein changes in small patient collectives, but inadequate for large-scale studies because the processing time for both microdissection and mass spectrometry is longer, and the cost is higher.

Moreover, due to tissue heterogeneity, single-protein-marker classification usually does not result in sufficient accuracy for clinical routine. Several previous studies described the fact that MALDI-MSI-derived *m/z* value (signatures) in combination with supervised machine learning models enable a robust cancer-tissue classification [28,47,48,49]. In previous studies, we used MALDI-MSI to distinguish among four different epithelial ovarian-cancer histotypes [50] to predict a proteomic signature in early-stage ovarian cancer disease, which is a prognostic marker for recurrence [51], and to classify molecular subtypes of high-grade serous ovarian cancer [28]

In the present study, we apply this technique in combination with neural-network strategies to expose spatially resolved proteomic-signatures for pancreatic-ductal-adenocarcinoma classification. As data size and the high dimensionality of MALDI-MSI analyses still pose complex computational and memory requirements that hinder highly accurate identification of relevant molecular patterns, we explore the feasibility of MALDI-MSI in combination with neuronal-network-evaluation strategies [52]. We demonstrate that machine learning tools, in particular neural networks, given high-dimensional MALDI-MSI data are able to identify ductal carcinoma, giving high-dimensional MALDI-MSI data with an accuracy of up to 86%. MALDI-MSI combined with machine learning enables an accurate and quick PDAC prediction of large data sets, with a minimum of data preprocessing.

Fast and robust prediction is needed to enable the integration of MALDI-MSI in the clinical workflow. However, most studies apply a two-step pipeline for data processing [53], consisting of feature selection and subsequent classification steps. Firstly, features are selected either by hand, using the well-known practice of peak selection [54], or using a dimension-reduction algorithm such as principal-component analysis or non-negative matrix factorization [27], before applying an—often linear—classifier or a thresholding approach. This data-processing pipeline is time consuming, and selecting peaks (features) to increase classification performance can result in potentially valuable data being discarded, thus resulting in negative robustness. Therefore, in this work, we make use of the non-processed spectra, and do not apply any type of explicit feature selection. Non-linear spectral diversity has the potential to determine biologically relevant clusters for tissue assessment and clinical phenotypes prediction.

Neural networks can use non-linear mapping to reveal correlations in the spectral data which are not accessible with the established linear methods. In addition, these methods enable the combination of the feature-selection and classification stage in an end-to-end fashion [49]. Our model architectures are well suited to deal with sequential data such as MALDI-MSI data.

Consequently, we directly apply neural networks to the raw spectrum-data, and utilize the inherent-feature-extraction capabilities, similar to in the work of Behrmann et al. in [49]. In our previous work, we demonstrated that a convolutional network with skip-connections can differentiate four different subtypes of ovarian cancer when applied to MALDI-MSI data [50]. Our implementation is based on the original ResNet architecture [55], which famously increased the performance on the famous ImageNet classification challenge [56] by a large margin. We also considered the Transformer architecture, which has gained increasing popularity in fields such as language translation or caption generation, and is one of the most effective tools for processing sequential data: MALDI-MSI-derived spectra can be seen as sequential data, as each mass peak is linked to a specific detection-time. As we are interested in classification rather than sequence-to-sequence transformation, we only employ the encoder part of the original Transformer’s encoder-decoder design, with an added classification layer.

The capability of the proposed methods to extract robust features from the given spectral data is limited by the extent of noise. These features are crucial for the subsequent classification steps. The MALDI-MSI acquisition technique, due to its high resolution, results in noisy data. This sensitivity to noise hinders the learning process. The problem of acquisition-related noise is described in [29], and can be compensated for, but the problem of noise due to structurally non-informative spectral data points remains. In our work, we implemented a filter to manage these data points. The proposed filter aims to reject noisy spectra before the classification step. Applying the filter to each spectrum results in a total of 2183 rejected spectra, due to their low informativeness, and improves classification by 3% across the models.

In total, our proposed method allows us to correctly classify ductal carcinoma with an accuracy of 86% and a sensitivity of 82%. The entire spectral data (full *m/z* range) can be used without time-consuming feature (*m/z*) selection. In upcoming studies, larger cohorts will be tested by a trained network to verify these findings. This will allow us to elevate our method from a classification algorithm to a more broadly applicable tool for diagnostic research.

## 5. Conclusions

Accurate and reliable diagnoses of pancreatic ductal adenocarcinoma (PDAC) are currently not adequately available. This pilot study demonstrates that (1) MALDI-MSI, combined with nanoLC-MS/MS, is a feasible pathway for identifying discriminative *m/z* values from corresponding proteins in pancreatic ductal adenocarcinoma versus ampullary carcinoma, which might have an important role in tumor progression, and (2) MALDI-MSI with neural-network strategies provides an accurate classification of PDAC without time-consuming feature-extraction methods such as peak picking. Moreover, we address the caveat of noisy data which is inherent to MALDI-imaging, utilizing only properties of the spectrum signal itself, without the need for user interaction. This study provides a proof-of-concept for the usefulness of the technology in assisting pancreatic-ductal-adenocarcinoma classification in a fast and cost-effective manner.

## Figures and Tables

**Table 1:** Clinicopathological characteristics of our patient cohort.

	Study Group	PDAC	AC	Other PC
Total *n*	446	260	103	83
Age at Surgery				
>60 years	311 (70%)	187 (72%)	74 (72%)	50 (60%)
<60 years	135 (30%)	73 (28%)	29 (28%)	33 (40%)
Sex				
female	211 (47%)	118 (45%)	45 (44%)	48 (58%)
male	235 (53%)	142 (55%)	58 (56%)	35 (42%)
Regional Lymph Nodes				
pN0: No regional lymph-node-metastasis	115 (26%)	64 (25%)	50 (49%)	1 (1%)
pN1: Regional lymph-node-metastasis	241 (54%)	180 (69%)	51 (50%)	10 (12%)
pNx: Cannot be assessed	90(20%)	16 (6%)	2(2%)	72 (87%)
Histologic Grade				
G1: Well differentiated	4 (1%)	3 (1%)	1(1%)	-
G2: Moderately differentiated	287 (64%)	181 (70%)	96 (93%)	10 (12%)
G3: Poorly differentiated	72 (16%)	65 (25%)	6 (6%)	1 (1%)
not assessed	94 (21%)	11 (4%)	-	72 (87%)

**Table 2:** Parameter configurations for network models.

Model	Kernel Size	Channel	Learning Rate	Batch Size	Heads	Pooling Size
Residual	(200, 100)	(16, 8)	1 × 10^−5^	100	-	-
Transformer 1	(256, 256)	(16, 8)	1 × 10^−4^	100	2	4
Transformer 2	(512, 512)	(16, 8)	1 × 10^−4^	500	2	8

**Table 3:** Dataset sizes after random split (percentage of total in parentheses).

	Training	Validation	Testing
Dataset 1	267 (51.1%) cores	100 (19.2%) cores	100 (19.2%) cores
	14,384 (50.3%) spectra	5578 (19.5%) spectra	8627 (30.2%) spectra
Dataset 2	270 (51.7%) cores	97 (18.6%) cores	155 (29.7%) cores
	14,323 (50.2%) spectra	5611 (19.7%) spectra	8619 (30.1%) spectra
Dataset 3	254 (48.7%) cores	102 (19.5%) cores	166 (31.8%) cores
	14,297 (50.1%) spectra	5577 (19.5%) spectra	8679 (30.4%) spectra

**Table 4:** Differential intensity distributions of *m/z* values (MALDI-MSI) and their corresponding proteins in tissue sections from pancreatic ductal adenocarcinoma and ampullary carcinoma tissue (tumor regions).

MALDI IMS *m/z* Value	ROC [AUC] AC/PDAC	LC-MS/MS [Mr+H^+^ cal.]	Deviation [Da]	MOWSE Score	Sequence	Protein	Gen Symbol
1459.7	0.721	1459.8631	−0.16	38.2	K.IGDLHPQIVNLLK.S	Collagen alpha-3(VI) chain	COL6A3
1586.8	0.700	1586.9265	−0.16	47.7	R.LQPVLQPLPSPGVGGK.R		
1267.7	0.715	1267.6529	0.01	53	K.AEGPEVDVNLPK.A	Neuroblast differentiation-associated protein AHNAK	AHNAK
1655.8	0.719	1654.8170	0.96	107.4	K.VDIEAPDVSLEGPEGK.L		
1461.7	0.710	1461.7366	−0.04	78.1	R.SQVMDEATALQLR.E	Plectin	PLEC
1479.8	0.714	1479.7914	−0.03	58.9	R.SLQEEHVAVAQLR.E		
2115.1	0.701	2115.0175	0.08	69.6	R.AGTLSITEFADMLSGNAGGFR.S		

**Table 5:** Results for neural-network models. Given are the model classification-accuracies for single spectra (spec) and full patient (sample) predictions.

Model	Split	Accuracy (Spec)	Accuracy (Sample)
Residual	I	0.86	0.86
	II	0.76	0.77
	III	0.77	0.76
		0.80	0.80
Transformer 1	I	0.85	0.86
	II	0.78	0.77
	III	0.77	0.77
		0.80	0.80
Transformer 2	I	0.83	0.84
	II	0.77	0.76
	III	0.78	0.79
		0.80	0.80

**Table 6:** Sensitivity and specificity metrics distinguished by histotype (PDAC and non-PDAC). Non-PDAC denotes all tumor and non-PDAC types labeled in the given data set. Reported are the results for all models.

Model	Class	Spot/Patient	Sensitivity	Specificity
Residual	PDAC	Spot	0.79	0.90
	Non-PDAC	Spot	0.90	0.79
	PDAC	Patient	0.82	0.90
	Non-PDAC	Patient	0.90	0.82
Transformer 1	PDAC	Spot	0.81	0.89
	Non-PDAC	Spot	0.89	0.81
	PDAC	Patient	0.83	0.88
	Non-PDAC	Patient	0.88	0.83
Transformer 2	PDAC	Spot	0.76	0.87
	Non-PDAC	Spot	0.87	0.76
	PDAC	Patient	0.78	0.89
	Non-PDAC	Patient	0.89	0.78

## Data Availability

Data is contained within the article or supplementary material. The MALDI-MSI data presented in this study are available on request from the corresponding author.

## Supplementary Materials

The following supporting information can be downloaded at https://www.mdpi.com/article/10.3390/cancers15030686/s1, Supplementary Figure S1: TMA dataset and split. Supplementary Figure S2: Example for spectra with low and high topology. Supplementary Table S1: Aligned *m/z* values from cell-rich tumor region in tumor microarray cores from AC and PDAC. Supplementary Table S2: Identified *m/z* values by using nanoLC-MS/MS.
