# Supplementary material for: Classification of Pancreatic Ductal Adenocarcinoma Using MALDI Mass Spectrometry Imaging Combined with Neural Networks
Source: Cancers (Basel). 2023 Jan 22;15(3):686. doi: 10.3390/cancers15030686 (PMC9913229; doi:10.3390/cancers15030686)
Supplement: Supplementary file 1 [file cancers-15-00686-s001.zip › cancers-2114053-supplementary/cancers-2114053-supplementary.docx]

Supplementary Figures

Classification of Pancreatic Ductal Adenocarcinoma Using MALDI-Imaging combined with Neural Networks

Frederic Kanter ^1^, Jan Lellmann^1^*, Herbert Thiele^7^, Steve Kalloger^2,^ David F. Schaeffer ^3,4^, Axel Wellmann^5^ and Oliver Klein^6,^

Supplementary Figure S1.

TMA dataset and split including (a) ground truth class labels, (b) exemplary randomized split ('Dataset 1') into 50% training data, (c) 20% validation data, and 30% test data. All classifiers were trained on the training and validation sets (b), (c) and evaluated on the previously unseen, disjoint test dataset (d).


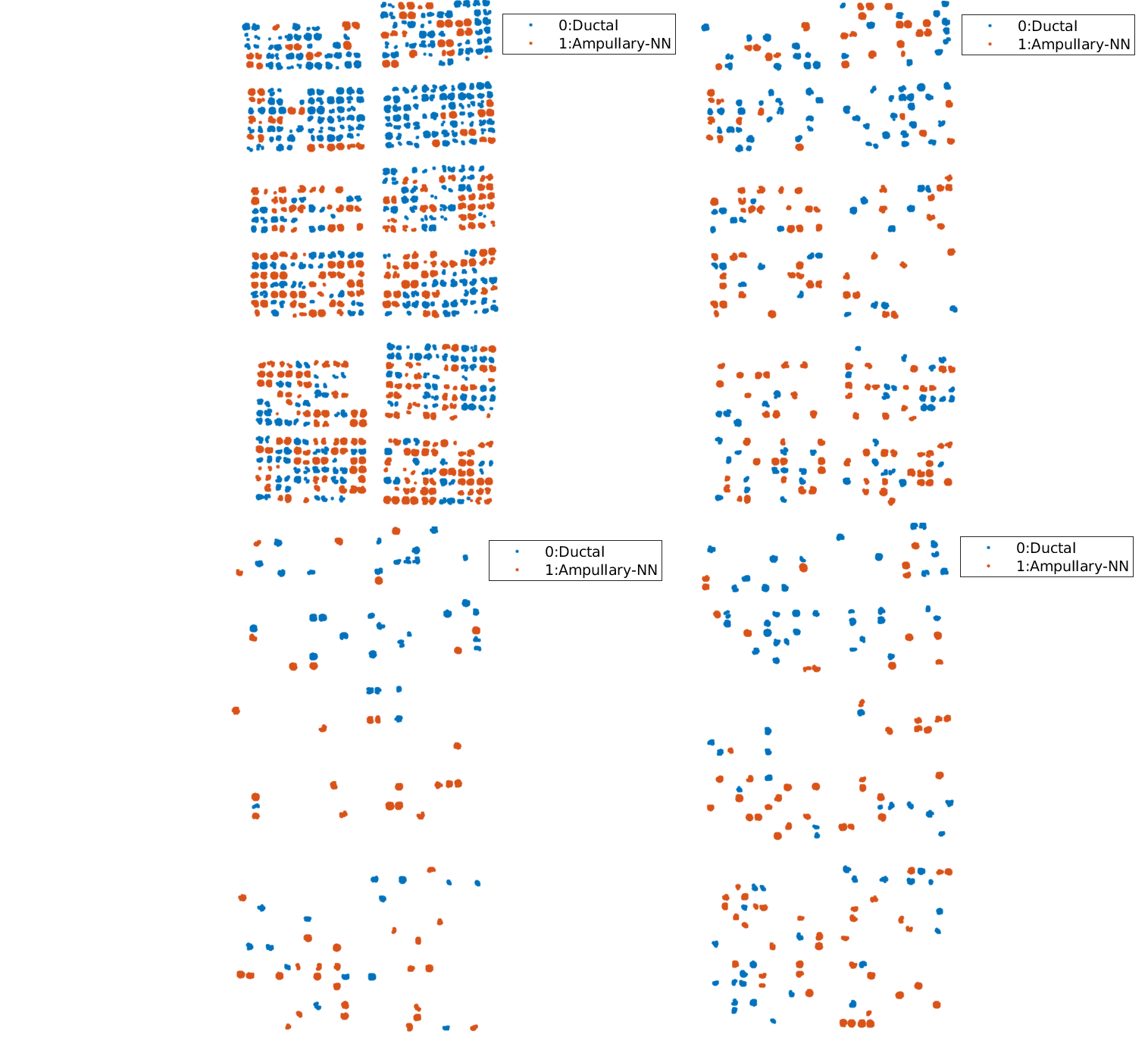


**a.**

**b.**

**c.**

**d.**

Supplementary Figure S2.

Example for spectra with low and high topology. (a): Spectrum with an informative topology. (b): Spectrum with a sufficient topology. (c): Spectrum with basically no topology. (d): Spectrum with an insufficient topology.

| (**a**) | (**b**) |
| --- | --- |

| 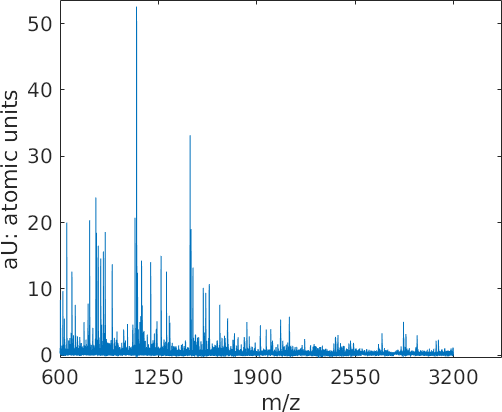 | 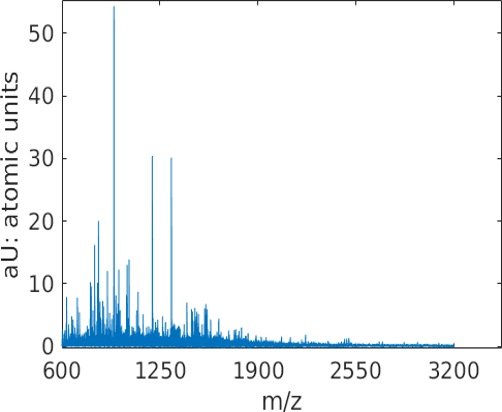 |
| --- | --- |
| (**c**) | (**d**) |
| 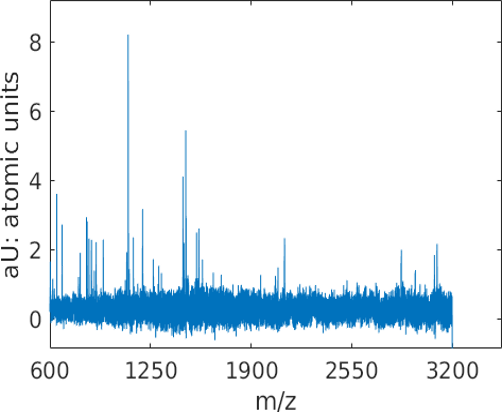 | 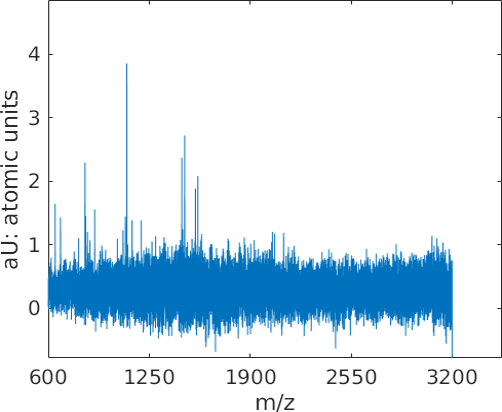 |
| (**c**) | (**d**) |

Supplementary Table S1: Aligned *m*/*z* values from cell-rich tumor region in tumor microarray cores from AC and PDAC. Supplementary Table S2: Identified *m*/*z* values by using nanoLC-MS/MS.
